# The Relaxant Effect of Crocin on Rat Tracheal Smooth Muscle and Its Possible Mechanisms

**DOI:** 10.22037/ijpr.2019.1100713

**Published:** 2019

**Authors:** Saeideh Saadat, Mahsa Yasavoli, Zahra Gholamnezhad, Mohammad Reza Aslani, Mohammad Hossein Boskabady

**Affiliations:** a *Neurogenic Inflammation Research Center, Mashhad University of Medical Sciences, Mashhad, Iran.*; b *Department of Physiology, School of Medicine, Mashhad University of Medical Sciences, Mashhad, Iran.*; c *Ardabil Imam Khomeini Educational and Clinical Hospital, Ardabil University of Medical Sciences, Ardabil, Iran.*

**Keywords:** Crocin, Smooth muscle, Trachea, Receptors, Adrenergic, Muscarinic

## Abstract

Crocin, a component of saffron, showed hypotensive activity which is perhaps due to vascular smooth muscle relaxant effect. The relaxant effects of saffron on tracheal smooth muscle also could be due to its constituent, crocin. In the present study, the relaxant effects of crocin and its possible mechanisms on rat tracheal smooth muscle were investigated. The relaxant effects of three cumulative concentrations of crocin (30, 60, and 120 μM) or theophylline (0.2, 0.4, 0.6 mM) as positive control were examined on pre-contracted tracheal smooth muscle by methacholine or KCl in non-incubated or incubated conditions with different agents including atropine, chlorpheniramine, indomethacin, diltiazem, glibenclamide, and propranolol. In non-incubated tracheal smooth muscle, crocin showed significant relaxant effects on KCl induced muscle contraction (*p* < 0.001 for two higher concentrations). However, crocin did not show relaxant effect on methacholine induced tissue contraction. In incubated tissues with chlorpheniramine, indomethacin, diltiazem and propranolol, there were no significant differences in the relaxant effects of crocin between non-incubated and incubated tissues. However, the relaxant effects of crocin obtained in incubated tissues with atropine and glibenclamide were significant lower than non-incubated tracheal smooth muscle (*p* < 0.05 to *p *< 0.001). The EC_50_ value obtained in incubated tissue with propranolol was significantly increased. Theophylline showed significant relaxant effect on both KCl and methacholine induced tissue contraction (*p* < 0.01 to *p* < 0.001). A relatively potent relaxant effect of crocin on tracheal smooth muscle, lower than that of theophylline was shown. Muscarinic receptor blocking, potassium channels opening and ß_2_-adrenoreceptors stimulation were also suggested as possible mechanisms of this effect.

## Introduction

The genus Crocus includes roughly 88 species among which *Crocus sativus* *L*. (*C. sativus*) or saffron, is the most important species which studied widely for its various pharmacological properties (1). Saffron, the dried stigma of the plant *C. sativus*, has three major characteristic components a) crocin, the principle coloring pigment (mono and diglycosyl esters of a polyene dicarboxylic acid, named crocetin), b) the glycoside picrocrocin which is a precursor of safranal and responsible for its distinctly bitter ﬂavor and c) safranal, a monoterpen aldehyde which is the deglycosylated form of picrocrocin and the major organoleptic principle of the stigmas (1).

The crocins (C_44_H_64_O_24_) are a group of hydrophilic carotenoides that are either mono- or di-glycosyl polyene esters of crocetin in which D-glucose and/or D-gentiobiose occur as carbohydrate residues.

At present, saffron is almost exclusively used as a natural ﬂavoring in the food industry. Recent studies have boosted interest in its medicinal properties as antioxidants (2-4), antitumorigenic (5-9), memory enhancers (10-13), antidepressants and anxiolytics (14-17), aphrodisiac (18-20), genoprotectives (21, 22), antitussives (23), cardioprotectives (24-27), and neuroprotectives (28, 29). The relaxant effect of the plant extract and safranal on tracheal smooth muscle (30, 31) and their effects on lung inflammation (32-35), pathological changes (32) and immunoregulation (34) in animal model of asthma were also shown.

The effects of saffron petals extracts on blood pressure, hypotensive effect of aqueous extract of *C. sativus* and its constituents, safranal and crocin, were shown (36). Administration of 50 mg/100 g of aqueous extract of saffron changed the blood pressure from 133.5 ± 3.9 to 117 ± 2.1 mmHg (36) and the effect of chronic administration of saffron stigma aqueous extract on systolic blood pressure was shown (37). In addition, the effect of saffron on uterine contraction was also investigated (38). For *C. sativus* and its constituent safranal, a stimulatory effect on ß_2_- adrenoceptors (31), an inhibitory effect on histamine H_1_ receptors (39) and a functional antagonistic effect on muscarinic receptors were demonstrated (40).

Abundant research has been conducted concerning the biological and pharmacological properties of two saffron ingredients, safranal (41) and crocin. It has been shown that crocin exhibits pharmacological effects on many organs including the nervous system, gastrointestinal, cardiovascular, genital, endocrine, immune systems, *etc* (42). Some of these pharmacological effects including learning and memory, depression and anxiety, cerebral ischemia via inhibition of reperfusion-induced oxidative/nitrative injury, atherosclerosis, hyperlipidemia and hypertension, myocardial injury, sexual function, carcinogenesis via delayed the formation of papillomas and strong cytotoxic effect, antioxidant activity, inflammation and genotoxicity (42). Hosseinzadeh and coworkers showed that crocin at pharmacological doses did not exhibit marked damages to all the major organs of the body (43). Crocin was shown Low toxicity in rats even in high experimental dosage (44).

The effects of chronic and subchronic crocin treatment in hypertension (45, 46) were demonstrated. Five weeks administration of three doses of crocin (50, 100 and 200 mg/kg/day) could reduce the mean systolic blood pressure (MSBP) in DOCA salt treated rats in a dose dependent manner. Crocin did not decrease the MSBP in normotensive rats (46). Although the last study did not show antihypertensive effect, this effect of crocin is perhaps due to its relaxant effect on vascular smooth muscle. The protective effect of crocin on reperfusion induced cardiac arrhythmias (47) as well as the lowering effect on heart rate and contractility was also documented (48). The results suggested that crocin is partially capable of suppressing reperfusion-induced arrhythmias (47). 

Therefore, in the present study, the relaxant effect of crocin on rat tracheal smooth muscle and the possible underlying mechanism(s) responsible for this effect were examined.

## Experimental

*Animals*

Sixty-four male Wistar rats weighing approximately 200–250 g (Animal house, School of Medicine, Mashhad University of Medical Sciences, Iran) were housed in Plexiglas cages in a temperature-controlled environment (20 ± 2 °C) on a 12-h light-dark schedule with standard diet and tap drinking water available *ad libitum*. The study was approved by the ethics committee of Mashhad University of Medical Sciences (No. 931653) for Animal Experiments.

*Tissue preparation*

For obtaining the trachea, the rats were sacrificed by blow on the neck and the chest was opened and excess of connective tissue and fat were dissected out. The trachea was cut into rings of 3 to 4 mm in width; each ring contains about four cartilages for the formation of tracheal ring. Each tracheal ring was hung between two Nichrome hooks inserted into the lumen, and placed in a 10 mL organ bath. The bath containing Krebs-Henseliet solution (KHS) composed of (mM): NaCl 120, KCl 4.72, KH_2_PO_4_ 1.2, MgSO_4_·7H_2_O 0.5, CaCl_2_·2H_2_O 2.5, NaHCO_3_ 25 and Dextrose 11 which was maintained at 37 ± 0.5 °C and bubbled constantly with 5% CO2-95% O2. Tissue was suspended under isotonic tension of 1 g and allowed to equilibrate for at least 1 h while it was washed with KHS solution every 15 min. In all experiments contraction responses were measured using an isotonic transducer (MLT0202, AD Instruments, Australia) which was connected to a power lab system (Power Lab 8/30, ML870, AD Instruments, Australia). The study duration was 12 months.

*Protocol*

Cumulative concentrations of crocin (30, 60, and 120 μM), purchased from Sigma Chemical Co (crocin-1, alpha-crocin, Crocetin di-β-D-gentiobiose ester, C_44_H_64_O_24_, molecular weight 976.972 g/mol, St Louis, MO, USA), or theophylline (0.2, 0.4, and 0.6 mM) as positive control (Sigma Chemical Co, St Louis, MO, USA) were added on pre-contracted tracheal smooth muscle in 5 min intervals to produce concentration response curves. As negative control, the effect of 1 mL saline on pre-contracted tracheal smooth muscle was also evaluated in each experiment. With regard to molecular weight of crocin (976.96 g/mol), the used studied concentrations of crocin are nearly 30, 60, and 120 μM. 

The percentage of relaxation due to each concentration of crocin or theophylline in proportion to the maximum contraction due to contractile agent (methacholine or KCl) was plotted against crocin or theophylline concentration to produce concentration response curve in each experiment. The effective concentration of crocin causing 50% of maximum response (EC_50_) was also calculated as previously described (41).

*Study groups*

To examine the relaxant effect of crocin, tracheal smooth muscle was contracted by contractile agent for 5 min on non-incubated or incubated tissues with different substance for 10 min as follows:

A- Tracheal smooth muscle was contracted by 60 mM KCl (Merk, Germeny) in the following groups;

I) Non-incubated tissues (n = 8)

II) Incubated tissues with:

a. One μM atropine (Sigma Chemical Ltd UK), (n = 7)

b. One μM chlorpheniramine (Sigma Chemical Ltd UK), (n = 7)

c. One μM indomethacin (Sigma Chemical Ltd UK), (n = 6)

d. Five μM diltiazem (Sigma Chemical Ltd UK), (n = 5)

e. One μM glibenclamide (Sigma Chemical Ltd UK), (n = 5)

f. One μM propranolol (Sigma Chemical Ltd UK), (n = 6)

B- Non-incubated tracheal smooth muscle contracted by 10 μM methacholine (Sigma Chemical Ltd. UK), (n = 8).

Each experimental group was done in random order in one tissues. Occasionally, few experimental groups were performed in one tissue with one hour resting period between two experiments while washing tissues with KHS solution every 15 min. The effect of theophylline as positive control was only examined on non-incubated tissues (n = 6 for each group). 

*Data analysis*

Data are presented as mean ± SEM. The results were analyzed using one-way analysis of variance (ANOVA) followed by Tukey’s Multiple comparison test. Statistically significant was considered as *p* < 0.05.

## Results

*The relaxant effect of crocin in non-incubated tracheal smooth muscle contracted with KCl*

**Figure 1:**
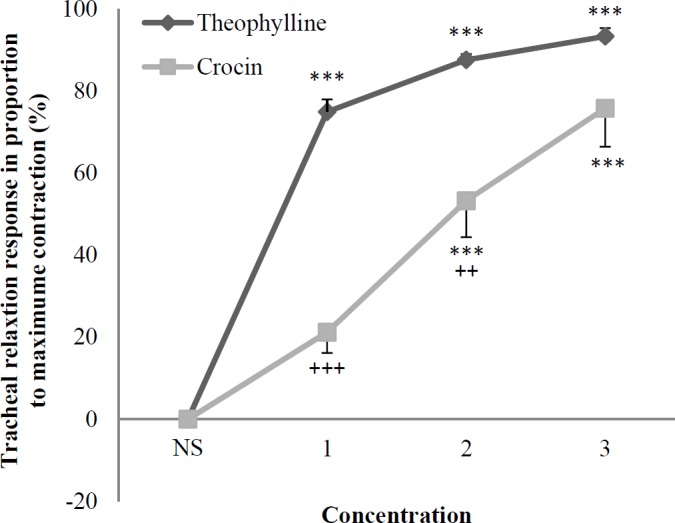
Concentration-response relaxant effect (mean ± SEM) of crocin (n = 8) and theophylline (n = 6) on KCl (60 mM) induced contraction of tracheal smooth muscle in non-incubated tissues. 1, 2 and 3 in X axis represent three concentration of crocin (30, 60, and 120 μM) and theophylline (0.2, 0.4, and 0.6 mM). ^***^p < 0.001 compared to saline (NS). ^++^*p *< 0.01, ^+++^*p* < 0.001 compared to the effect of theophylline. Statistical comparisons were performed using ANOVA with Tukey Kramer post-test

The two higher concentrations of crocin showed significant and concentration-dependent relaxant effects in non-incubated tracheal smooth muscle contracted by KCl (*p* < 0.001 for both cases), (Figure 1).

*The relaxant effect of crocin in incubated tracheal smooth muscle contracted with KCl*

**Figure 2:**
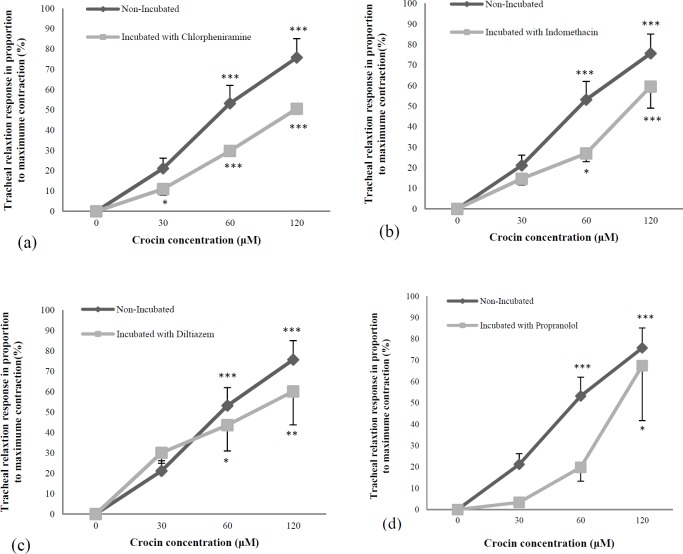
Concentration-response relaxant effect (mean ± SEM) of crocin on KCl (60 mM) induced contraction of tracheal smooth muscle in non-incubated (n = 8) and incubated tissues with (a) atropine (1 μM, n = 7), (b) glibenclamide (1 μM, n = 5). ^*^p < 0.05, ^***^p < 0.001, compared to saline (as indicated by zero in X axis of the figure). ^+^*p < *0.05, ^++^*p < *0.01 ^+++^*p < *0.001, compared to non-incubated tissues. Statistical comparisons were performed using ANOVA with Tukey Kramer post-test

Crocin showed significant and concentration-dependent relaxant effect in incubated tracheal smooth muscle with chlorpheniramine, indomethacin (*p < *0.05 to *p < *0.001 for both cases), diltiazem (*p < *0.05 to *p < *0.01), and propranolol (*p < *0.05), (Figure 2). There was no significant difference in the relaxant effects of crocin between non-incubated and incubated tissue with chlorpheniramine, indomethacin, diltiazem, and propranolol (Figure 2).

**Figure 3:**
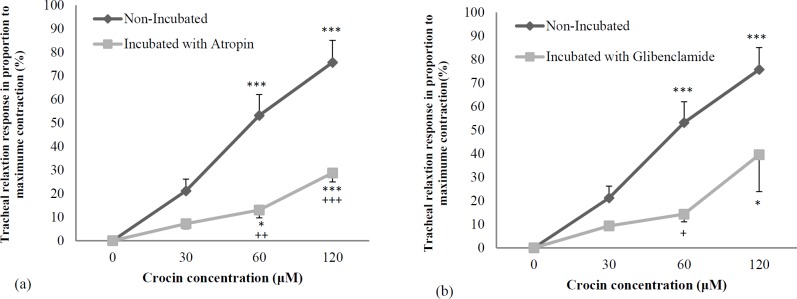
Concentration-response relaxant effect (mean ± SEM) of crocin on KCl (60 mM) induced contraction of tracheal smooth muscle in non-incubated (n = 8) and incubated tissues with (a) chlorpheniramine (1 μM, n = 7), (b) indomethacin (1 μM, n = 6), (c) diltiazem (5 μM, n = 5), and (d) propranolol (1 μM, n = 6).^ *^p < 0.05, ^**^p < 0.01, ^***^p < 0.001 compared to saline (as indicated by zero in X axis of the figure). Statistical comparisons were performed using ANOVA with Tukey Kramer post-test

In incubated tissues with atropine and glibenclamide, crocin showed significant and concentration-dependent relaxant effects (*p < *0.05 to *p < *0.001 and *p < *0.05 to *p < *0.01, respectively), (Figure 3). The relaxant effects of two higher concentrations of crocin were significantly lower in incubated tissue with atropine compared to non-incubated tracheal smooth muscle (*p < *0.01 to *p < *0.001), (Figure 3a). The relaxant effects of medium concentrations of crocin were significantly lower in incubated tissue with glibenclamide compared to non-incubated tracheal smooth muscle (*p < *0.05), (Figure 3b).

**Figure 4:**
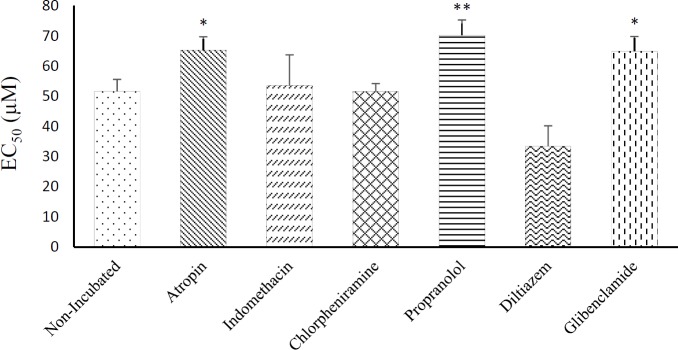
EC_50_ values of crocin induce relaxation obtained on contracted tracheal smooth muscles of rat with 60 mM KCl in non-incubated (n = 8) and incubated tissues with atropine (n = 7), chlorpheniramine (n = 7), indomethacin (n = 6), diltiazem (n = 5), glibenclamide (n = 5) and propranolol (n = 6). ^*^p < 0.05, ^**^p < 0.01 compared to non-incubated tissues. Statistical comparisons were performed using ANOVA with Tukey Kramer post-test

There was no significant difference in EC_50_ values of crocin between non-incubated (51.62 ± 4.01) and incubated tissue with chlorpheniramine (51.57 ± 2.67), indomethacin (53.5 ± 10.24), and diltiazem (33.4 ± 6.8). However, the results showed significant difference in EC_50_ values of crocin, between non-incubated and incubated tissue with atropine (65.28 ± 4.5), glibenclamide (65 ± 4.85), and propranolol (70.33 ± 5.01), (Figure 4).

The relaxant effect of low concentrations of crocin in diltiazem incubated tracheal smooth muscle was significantly higher than those of incubated tissues with atropine (*p < *0.001), chlorpheniramine (*p < *0.001), indomethacin (*p < *0.05), glibenclamide (*p < *0.001) and propranolol (*p < *0.001) in KCl induced muscle contraction. Additionally, the relaxant effect of medium concentrations of crocin in diltiazem incubated tissues was also significantly higher than those of incubated tracheal smooth muscle with atropine (*p < *0.01) and glibenclamide (*p < *0.05), (Table 1). The relaxant effects of medium and high concentrations of crocin in incubated tissues with atropine were significantly higher than those of incubated smooth muscles with chlorpheniramine and indomethacin (*p < *0.05 to *p < *0.01), (Table 1).

*The relaxant effect of crocin in non-incubated tracheal smooth muscle contracted by methacholine*

**Figure 5:**
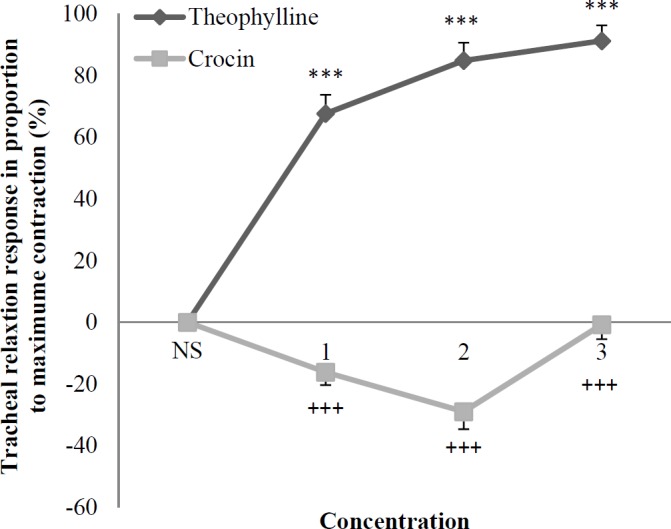
Concentration-response relaxant effect (mean ± SEM) of crocin (n = 8) and theophylline (n = 6) on methacholine (10 μM) induced contraction of tracheal smooth muscle in non-incubated tissues. 1, 2 and 3 in X axis represent three concentration of crocin (30, 60, and 120 μM) and theophylline (0.2, 0.4, and 0.6 mM). ^***^p < 0.001 compared to saline (NS). ^+++^*p < *0.01 compared to the effect of theophylline. Statistical comparisons were performed using ANOVA with Tukey Kramer post-test

In non-incubated tracheal smooth muscle contracted by methacholine, crocin did not show any relaxant effect (Figure 5).

**Figure 6:**
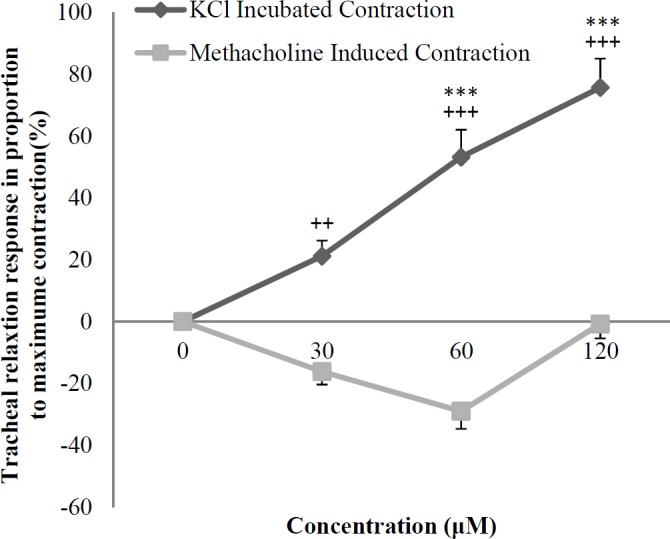
Concentration-response relaxant effect (mean ± SEM) of crocin on methacholine (10 μM) and KCl (60 mM) induced contraction of non-incubated tracheal smooth muscle (n = 8). ^***^p < 0.001 compared to saline (as indicated by zero in X axis of the figure). ^++^*p < *0.01, ^+++^*p < *0.001, compared to the relaxant effect on methacholine induced muscle contraction. Statistical comparison of the effect of each concentration between two groups was performed using unpaired *t*-test

The relaxant effects of various concentrations of crocin on tracheal smooth muscle contracted by methacholine were significantly lower than those obtained in KCl induced contraction (*p < *0.01 to *p < *0.001), (Figure 6).

*Comparison of the relaxant effect of crocin and theophylline*

The relaxant effect of two lower concentrations of crocin was significantly lower than that of theophylline in KCl induced contraction of non-incubated tracheal smooth muscle (*p < *0.01 to *p < *0.001), (Figure 1). 

The relaxant effects of all concentrations of theophylline were significantly higher than those of crocin in tracheal smooth muscle contracted by methacholine (*p < *0.001 for all concentrations), (Figure 5).

## Discussion

In this study, the relaxant effect of crocin in pre-contracted tracheal smooth muscle by KCl and methacholine was examined. In non-incubated tracheal smooth muscle contracted by KCl, crocin showed significant and concentration dependent relaxant effect. The relaxant effect of two higher concentrations of crocin was significantly lower than that of theophylline. These results indicated a relatively potent relaxant effect of crocin.

For *C. sativus* and its constituent safranal, a stimulatory effect on ß_2_- adrenoceptors (31), an inhibitory effect on histamine H_1_ receptors (47) and a functional antagonistic effect on muscarinic receptors were demonstrated (48). To evaluate these possible mechanisms, as well as other possible mechanisms for the relaxant effect of crocin, its effect was examined on tracheal smooth muscle incubated with atropine, chlorpheniramine, indomethacin, diltiazem, glibenclamide, and propranolol.

In incubated tracheal smooth muscle with atropine and contracted with KCl, the relaxant effect of crocin examined to assess the contribution of muscarinic receptor inhibitory effect on the relaxant property of crocin. The results showed significant lower relaxant effects of two higher concentrations of crocin in incubated tissues with atropine compared to non-incubated tracheal smooth muscle. These results indicated the inhibitory effect of crocin on muscarinic receptors which could be contributed in its relaxant effect on tracheal smooth muscle. In fact, the relaxant effect of muscarinic receptor blocking drugs on tracheal smooth muscle was previously documented (49). The study of Neamati *et al.* (2010) demonstrated the functional antagonistic effect of *C. sativus* and safranal on muscarinic receptors on tracheal muscle of guinea pigs. Both the extract and safranal shifted methacholine concentration-response curve to the right. However, the shift was not parallel and the maximum response to methacholine in the presence of extract and safranal was not obtained. These results indicated a functional antagonistic effect of the plant and safranal on muscarinic receptors (48). These two studies nearly supported the results of the present study regarding the inhibitory effect of crocin on muscarinic receptors. 

To examine the effect of crocin on histamine (H1) receptor and the role of this mechanism on the relaxant effect of crocin on tracheal smooth muscle, the relaxant effect of crocin was examined on tracheal smooth muscle incubated with chlorpheniramine and contracted with KCl. The findings of this group showed non-significant difference in the relaxant effect of crocin in incubated tracheal smooth muscle with the results of non-incubated tissue. These results indicate the absence of the inhibitory effect of crocin on histamine (H1) receptor and therefore, this mechanism is not responsible for the relaxant effect of crocin on tracheal smooth muscle. However, the relaxant effect of histamine (H1) receptor blocking drugs on tracheal smooth muscle was shown previously (50). The inhibitory effect of extracts of *C. sativus* on histamine (H1) was also shown previously. The effect of three concentrations of aqueous-ethanolic extracts of *C. sativus* (0.025%, 0.05% and 0.1%) on histamine (H1) receptors was evaluated in guinea pig tracheal smooth muscles previously. Concentration-response curve of histamine was obtained in the presence of saline, saffron extract, and chlorpheniramine. The extract caused parallel rightward shift in histamine concentration-response curve similar to the effect of chlorpheniramine and the maximum response to histamine was obtained in the presence of the extract. These results showed an inhibitory effect of *C. sativus* (competitive antagonistic effect) on histamine H1 receptors which could be related to the relaxant effect of the plant on tracheal smooth muscle (47). The reason of the differences between the effect of the extract of saffron, safranal and corcin on histamine (H1) receptors is uncertain to us and should be examined in further studies. 

To evaluate the involvement of prostacyclin mechanism in crocin induced tracheal smooth muscle relaxation, its effect was also examined in incubated tissues with indomethacin. Prostacyclin (PGI2) is an epithelium releasing factor produced by epithelial cyclooxygenase. Prostacyclin leads to elevation of cyclic AMP and finally reduces the availability of calcium and induces smooth muscle cell relaxation (51). The anti-inflammatory effect of crocin was also seen previously (52). Non-significant difference in the relaxant effect of crocin between non-incubated and incubated tissues with indomethacin showed that COX inhibitory effect of crocin does not affect its tracheal smooth muscle relaxation.

In incubated tracheal smooth muscle with diltiazem also the relaxant effect of crocin was studied to assess the role of calcium channel-blocking property in the relaxant effect of crocin. No significant difference was seen between the relaxant effects of crocin in non-incubated and incubated tissue with diltiazem. Therefore, calcium channel-blocking is not contributed in the relaxant effect of crocin on tracheal smooth muscle. Crocin could inhibit Ca^2+^ influx and release of intracellular Ca^2+^ stores in the endoplasmic reticulum in bovine aortic smooth muscle cells (53). It was also shown that reduction of intracellular Ca^2+^ release may contribute to relaxation of the corpus cavernosum, leading to erection (54). The effect of *C. sativus* on Ca^2+ ^influx in isolated rat aortas was investigated using 45Ca as a radioactive tracer, and their calcium antagonistic effects were evaluated. Ca^2+^ uptake in isolated rat aorta rings in normal physiological status was not markedly altered by these drugs, whereas Ca^2+^ influxe induced by norepinephrine 1.2 µmol/L and KCl 100 mmol/L were significantly inhibited by *C. sativus* in a concentration-dependent manner. The results showed that Ca^2+^ influx through receptor-operated Ca^2+^ channels and potential-dependent Ca^2+^ channels can be blocked by the plant (55). It has been reported that crocetin decreased protein kinase C (PKC) activity in the membrane fraction, which led to reduced blood pressure by inhibition of proliferation in vascular smooth muscle cells (56). However, the results of the present study did not reveale a calcium channel inhibitory effect of crocin. The possible reason of the differences between the results of the and previously studies should be examined in further studies.

**Table 1:** Comparison of the relaxant effects of three concentrations of crocin (percentage change in proportion to the maximum contraction) in different incubated tracheal smooth muscles contracted by 60 mM KCl

**Incubating substance**	**Concentration (μM)**		
	**30**	**60**	**120**
Atropine	7.24 ± 2.21***	13.03 ± 3.28**	28.8 ± 3.79
Chlorpheniramine	11.05 ± 3.1***	29.78 ± 2.2##	50.55 ± 2.68#
Indomethacin	14.69 ± 3.07 * +	27.01 ± 4.04#	59.61 ± 10.55##
Diltiazem	30.08 ± 5.16	43.65 ± 12.67	60.21 ± 16.42
Glibenclamide	9.32 ± 1.76***	14.3 ± 3.26*	39.56 ± 15.71
Propranolol	3.37 ± 0.82***	19.81 ± 6.51	67.44 ± 25.71

Data were presented as mean ± SEM. *p < 0.05, **p < 0.01, ***p < 0.001 compared to incubated tissues with diltiazem. +*p < *0.05 compared to incubated tissues propranolol. #*p < *0.05, ##*p < *0.01 compared to incubated tissues with atropine.

In incubated tracheal smooth muscle with glibenclamide as well as the relaxant effect of crocin was studied to assess the role of potassium channel-blocking property in its relaxant effect. The relaxant effects of medium concentrations of crocin were significantly lower in incubated tissue with glibenclamide compared to non-incubated tracheal smooth muscle. These results indicated the inhibitory effect of crocin on potassium channels which could be contributed in its relaxant effect on tracheal smooth muscle.

The most possible mechanism for the relaxant effect of agents on tracheal smooth muscle is their stimulatory effect on ß_2_-adrenergic receptors. To evaluate the effect of crocin on ß_2_- adrenoceptors and the role of this mechanism, the relaxant effect of crocin was examined on tracheal smooth muscle incubated with propranolol and contracted with KCl. The relaxant effect of ß_2_- adrenoceptors stimulatory drugs on tracheal smooth muscle was shown previously (50). There was no significant difference in the relaxant effects of crocin between non-incubated and incubated tissue with propranolol. These results indicate the absence of the stimulatory effect of crocin on ß_2_- adrenoceptors and therefore, this mechanism is not responsible for the relaxant effect of crocin on tracheal smooth muscle. However, the ß_2_-adrenergic stimulatory effect of the plant and safranal was tested by performing cumulative concentration-response curves of isoprenaline-induced relaxation of pre-contracted isolated guinea pig tracheal smooth muscle. The results showed leftward shifts in isoprenaline curves obtained in the presence of saffron extract and safranal compared to that of saline while propranolol caused rightward shift in isoprenaline response curve. The results indicated a relatively potent stimulatory effect of *C. sativus* extract and its constituent safranal on ß_2_-adrenoreceptors (31). Therefore, the results suggested that the major mechanism responsible for the relaxant effect of the plant and safranal is their stimulatory effect on ß_2_-adrenoreceptors. This discrepancy also should needs further investigations. 

Higher EC_50_ values of crocin induced relaxant effect and lower relaxant effect of crocin obtained in incubated tissues with atropine and glibenclamide compared to non-incubated tissues also support the contribution of muscarinic receptor inhibitory and potassium channel-blocking properties of crocin in its relaxant effect on tracheal smooth muscle. The results also showed significant difference in EC_50_ values of crocin between non-incubated and incubated tissue with propranolol. Although, there was no significant difference in the relaxant effects of crocin between non-incubated and incubated tissue with propranolol, the higher EC_50_ value obtained in incubated tissues with propranolol may indicate a component of stimulatory effect of crocine on ß_2_-adrenoreceptors.

On methacholine induced contraction of tracheal smooth muscle, crocin did not show any significant relaxant effect. The absence of the relaxant effect of crocin in the methacholine induced contraction of smooth muscle almost excluded the role of the muscarinic receptor inhibitory effect of crocin on its smooth muscle relaxant property. The discrepancy between the results of crocin on methacholine induced muscle contraction and incubated tissue with atropine and contracted with KCl is unclear to us and should be evaluated in further studies.

The absence of the relaxant effect of crocin on methacholine induced muscle contraction and relatively potent relaxant on KCl induced muscle contraction may indicate that the main mechanism of the relaxant effect of crocin is its inhibitory effect on calcium channels and/or opening effect on potassium channels. In fact, the relaxant effect of potassium channels opening drugs (57-59) and calcium channel blocking drugs (60) on tracheal smooth muscle were shown previously.

Previous studies showed the relaxant effect of crocin in various types of smooth muscle which support the findings of the present study. Imenshahidi *et al.* compared the hypotensive effect of saffron aqueous extract and its two active ingredients in rats. Based on their results, aqueous extract of saffron stigma, safranal and crocin decreased mean arterial blood pressure in a dose-dependent manner. The hypotensive effect of the extract is perhaps due to its relaxant effect on vascular smooth muscle. The results also suggested that safranal, the major constituent of the plant, contributes to the hypotensive activity (25). The effect of crocin (50 mg/kg) on the reduction of systolic blood pressure (SBP) and the increased heart rate (HR) induced by diazinon (DZN) in rats, was shown which could be due to the relaxant effect of crocin on vascular muscle cells (38). It was also shown that crocetin (15, 30 mg/kg) dose-dependently improved endothelium-dependent relaxation (EDR) in response to acetylcholine (Ach) in high cholesterol diet (HCD)-fed rabbits. In addition, in bovine aortic endothelial cells (BAECs), oxidized LDL (oxLDL) treatment decreased nitric oxide production and down-regulated the activity and mRNA expression of endothelial nitric oxide synthase and these effects were inhibited by crocetin (0.1, 1, 10 mM) in a dose-dependent manner (61). The effect of *C. sativus* petals extracts on isolated guinea-pig ileum induced by electrical field stimulation (EFS) was studied. In rat isolated ileum, contractile responses to EFS were decreased by the petals extracts. Contractions of ileum to EFS are mediated by both noradrenaline and ATP released as co-transmitters from sympathetic nerves (36). The discrepancy regarding the possible mechanisms of the relaxant effect of crocin on smooth muscle observe in the above study and the current study may be due to the types of smooth muscles (caprine detrusor muscle vs tracheal) and the distribution of various receptor and channels in different types of smooth muscle.

Although the concentrations of thephylline and crocin were not the same, their concentrations were chosen according the previous studies (30, 45). In addition, theophylline was used as a positive control in the present study as previous studies. Therefore, the effects of high, medium, and low concentrations of crocin were compared to the effect of corresponding concentrations of theophylline. 

In conclusion, the present study has demonstrated a relatively potent relaxant effect of crocin on tracheal smooth muscle which was lower compared to the effect of theophylline at studied concentration. The ﬁndings also suggested that the possible mechanisms of the relaxant effect of the crocin on tracheal smooth muscle including muscarinic receptor blocking, potassium channels opening, and ß_2_-adrenoreceptors stimulation.
